# Intestinal Dendritic Cells Are Altered in Number, Maturity and Chemotactic Ability in Fulminant Hepatic Failure

**DOI:** 10.1371/journal.pone.0166165

**Published:** 2016-11-10

**Authors:** Mei Liu, Peng Wang, Min Zhao, DY Liu

**Affiliations:** 1 Medical Research Center, Shengjing Hospital of China Medical University, Shenyang City, Liaoning Province, China; 2 The second department of urology, Shengjing Hospital of China Medical University, Shenyang City, Liaoning Province, China; Shanghai Jiao Tong University School of Medicine, CHINA

## Abstract

Fulminant hepatic failure (FHF) is defined as rapid acute liver injury, often complicated with spontaneous bacterial peritonitis (SBP). The precise onset of FHF with SBP is still unknown, but it is thought that SBP closely correlates with a weakened intestinal barrier. Dendritic cells (DCs) play a crucial role in forming the intestinal immune barrier, therefore the number, maturity and chemotactic ability of intestinal DCs were studied in FHF. Mouse intestinal and spleen DCs were isolated by magnetic-activated cell sorting (MACS) and surface markers of DCs, namely CD11c, CD74, CD83 and CD86, were identified using flow cytometry. Immunohistochemistry and Western blotting were performed to detect the distribution and expression of CC-chemokine receptor 7 (CCR7) and CC-chemokine receptor 9 (CCR9), as well as their ligands-CC-chemokine ligand 21 (CCL21) and CC-chemokine ligand 25 (CCL25). Real-time PCR was used to detect CCR7 and CCR9 mRNA, along with their ligands-CCL21 and CCL25 mRNA. Flow cytometry analysis showed that the markers CD74, CD83 and CD86 of CD11c^+^DCs were lower in the D-galactosamine (D-GalN) group and were significantly decreased in the FHF group, while there were no significant changes in the expression of these markers in the lipopolysaccharide (LPS) group. Immunohistochemistry results showed that staining for CCR7 and CCR9, as well as their ligands CCL21 and CCL25, was significantly weaker in the D-GalN and FHF groups compared with the normal saline (NS) group or the LPS group; the FHF group even showed completely unstained parts. Protein expression of CCR7 and CCR9, as well as their ligands- CCL21 and CCL25, was also lower in the D-GalN group and decreased even more significantly in the FHF group. At the gene level, CCR7 and CCR9, along with CCL21 and CCL25 mRNA expression, was lower in the D-GalN group and significantly decreased in the FHF group compared to the NS and LPS groups, consisting with the protein expression. Our study indicated that intestinal DCs were decreased in number, maturity and chemotactic ability in FHF and might contribute to a decreased function of the intestinal immune barrier in FHF.

## Introduction

Fulminant hepatic failure (FHF) refers to sudden necrosis of liver cells and fast deterioration of liver functions. Hepatic encephalopathy often occurs in patients with an outbreak with FHF[1]. Multiple organ damage may be caused by FHF. Spontaneous bacterial peritonitis(SBP), which is among the most severe complications arising in patients with FHF, is a frequent, life-threatening bacterial infection in patients with liver cirrhosis and ascites [2]. Increased bacterial translocation from the intestine caused by portal hypertension [2], and small-intestinal bacterial overgrowth (SIBO) is highly prevalent in patients with cirrhosis [3]. SBP is recognized as an important marker of liver disease progression, and could be the decisive turning point in the management of advanced liver disease [4]. The mechanisms underlying FHF concurrent with SBP are not yet fully understood. Decreased intestinal barrier functions have been proved in animal models and humans in advanced liver disease [5, 6]. Previous studies have shown that intestinal IgA, secretory component (SC) and secretory immunoglobulin A (SIgA) tend to be markedly decreased in patients with FHF [7]. Dendritic cells (DCs) are sentinel immune cells in the intestinal immune barrier and charging of initiating and polarizing adaptive immune responses [8]. In steady-state conditions, resident DCs express low levels of the costimulatory molecules-CD80 and CD86, as well as low levels of the major histocompatibility complex (MHC) class II to become phagocytic cells. Upon activation by microbes, DCs upregulate MHC and costimulatory molecules at the cell surface to downregulate phagocytic activity while increase processing capacity[9]. After loading with mucosal antigens, DCs migrate to the mesenteric lymph nodes(MLNs) to present the processed antigen to naive T cells. CC-chemokine receptor 7 (CCR7) and its ligand CCL21 are widely recognized as the most important driver of DC trafficking from peripheral tissues to draining lymph nodes [10, 11]. CC-chemokine receptor 9 (CCR9) is another candidate chemokine receptor for the regulation of DCs trafficking; the interaction between CCR9 and its ligand CCL25 contributes to the migration of T cells and DCs into the small intestine and movement of T cells in the thymus [12].

The model of FHF or acute liver injury induced by lipopolysaccharide (LPS) and D-galactosamine (D-GalN) has been widely used. Endotoxin is a gram-negative bacterial LPS and releases a wide variety of inflammatory mediators, which lead to necrosis of liver cells and liver injury. D-GalN is hepatotoxic agent that causes irreversible damage to liver cells by consuming uridine nucleotides in hepatocytes. Combined administration LPS and D-GalN leads to acute injury of liver cells and mimics the situations in FHF. We recently showed that CD11b/c, CD83, CD86 and the MHCⅡ-associated invariant chain Ii (also known as CD74), the T cell marker (CD3), and AKT/phosphorylated-AKT (p-AKT) were significantly altered in FHF[13]. The aim of our study was to investigate the changes in maturity and chemotaxis of DCs in FHF to further reveal the dysfunction of the intestinal immune barrier and provide a theoretical basis for FHF complicated with SBP.

## Material and Methods

All animals in this study were provided by the Animal Center of Shengjing Hospital of China Medical University. Adult wild-type mice were anesthetized and killed by cervical dislocation. All studies were performed in accordance with the protocol approved by the Institutional Animal Care and Use Committee of the China Medical University for Basic Research in Developmental Disabilities. All surgery was performed under anesthesia, and all efforts were made to minimize suffering.

### Animal model of FHF

A mouse model of FHF was established as described previously [7, 13, 14]. A total of 180 Male BALB/c mice weighing 18–22 g were randomly divided into four groups, namely group 1 (20 mice): normal saline (NS); group 2 (40 mice): LPS; group 3 (40 mice): D-GalN; and group 4 (80 mice): FHF (LPS and D-GalN; Sigma, USA). Mice were injected intraperitoneally with LPS (10 μg/kg) and/or D-GalN (800 mg/kg). Mice were killed at 9 h after the injection. The study was approved by the animal Ethics Committee of China Medical University.

### Isolation of intestinal CD11c^+^DC

Mice were sacrificed by cervical dislocation, and the entire small intestine luminal contents were flushed with PBS; the tissues were dissected longitudinally, cut into 1 cm pieces, and washed extensively in HEPES-buffered HBSS (Hyclone Technologies, USA). The small intestine pieces were incubations for 20 min at 37°C with continuous agitation in Ca^2+^Mg^2+^-free HBSS (Hyclone Technologies, USA) containing 10% FBS, 10 mM HEPES, and 5 mM EDTA for removal of epithelial cells. Then the small intestine pieces were digested in RPMI 1640 (Hyclone Technologies, USA) supplemented with 1mg/ml collagenase type IV (Sigma Aldrich, USA), 50μg/ml DNaseI, 10% FBS (Hyclone Technologies, USA), 2 mM-glutamine, 10mM HEPES, and 100g/ml gentamicin (collagenase-supplemented RPMI 1640) for 50min at 37°C with continuous agitation. Single cell suspension was obtained by passing through 100-mesh nylon sieve. 40%/70% Percoll (Amersham Biosciences, USA) density gradient centrifugation was used for further purified. DC-enriched cell populations at the interface were collected, washed with PBS and incubated with CD11c microbeads (Miltenyi Biotec, Germany) according to the manufacturer’s instructions. CD11c^+^ cells purification were conducted by positive selection using MACS separation columns (Miltenyi Biotec, Germany).

### Isolation of spleen CD11c^+^DCs

Mice were sacrificed by cervical dislocation. Spleens were removed, any fatty tissue was trimmed away and spleens were washed three times with PBS. Spleens were laid on a 100-mesh pore size cell strainer and, using the barrel from a 2mL syringe, the spleens were pressed through the strainer until only a small amount of fibrous tissue remained in the strainer. Spleen cells were collected and further purified using a mouse spleen lymphocyte isolation kit (TBD Sciences, China). Spleen DCs were isolated by MACS in a manner similar to isolation of small intestine DCs described above.

### Flow cytometry analysis

CD11c^+^DCs were incubated at 4°C for 30 minutes with the following fluorochrome-labeled anti-mouse monoclonal antibodies: anti-CD11c-APC (Biolegend, USA), anti-CD74-FITC (BD Biosciences, USA), anti-CD83-PE (Biolegend, USA) and anti-CD86-PE (Biolegend, USA). Samples were acquired on a FACS Calibur flow cytometer (BD Biosciences, USA) and data were analyzed with Flowjo software (BD Biosciences, USA).

### Immunohistochemical analysis

Small intestine tissues were fixed in 4% paraformaldehyde, embedded in paraffin, and cut into 3.5μm slices. Followed deparaffinized, rehydrated and antigen retrieval, endogenous peroxidase activitywas blockedwith 3% H_2_O_2_. Primary antibodies of CCR7 (Abcam, USA), CCR9 (Abcam, USA), CCL21 (R&D Systems, USA), and CCL25 (R&D Systems, USA) were incubated at 4°C overnight. After washing, the slides were incubated with apporiate biotin-labeled secondary antibodies followed by staining with 3, 3’-diaminobenzidine (DAB), and counterstaining with hematoxylin. The stained slides were examined by light microscopy.

### Western blot assay

Small intestinal tissues were lysed in RIPA buffer. Equal amount of protein samples (40μg) were separated by 10% and 12% SDS-PAGE, and then transferred to polyvinylidene fluoride membranes (Millipore, USA). Membranes were blocked in 5% non-fat milk dissolved in TBST-Tween 20, followed by incubated with specific primary antibodies-CCR7 (Abcam, USA), CCR9 (Abcam, USA), CCL21 (R&D Systems, USA), CCL25 (R&D Systems, USA) and GAPDH (Abcam, USA) overnight at 4°C, respectively. The next day, membranes were incubated with HRP-conjugated rabbit anti-mouse IgG (ZSGB-BIO, China) and goat anti-rabbit IgG (ZSGB-BIO, China). All the immunoblots were visualized by using an ECL detection system (Thermo, USA).

### Real-time polymerase chain reaction (PCR) analysis

Total RNA from murine small intestines was extracted with TRIzol^TM^ (Takara Biotechnology, China) and cDNA (Takara Biotechnology, China) was synthesized using PrimeScript^®^ RT reagent Kit (Takara Biotechnology, China) according to the manufacturer’s guidelines. Quantitative real-time PCR was performed using the LightCycler® 480 Real-Time PCR System (Roche Applied Science, Germany). The sequences of the forward and reverse primers were used to amplify gene-specific regions:5’-CTCTCCTTGTCATTTTCCAGGTGTG-3’ and 5’-ACACCGACTCGTACAGGGTG-3’ for CCR7, 5’-TGTAAGAAAAATAATGTCAGGCAGT-3’ and 5’-CAGAAGGGAAGAGTGGCAAG-3’ for CCR9, 5’-CGGCAATCCTGTTCTCACCC-3’ and 5’-GGAGCCCTTTCCTTTCTTTC-3’ for CCL21, 5’-TGGGTTACCAGCACAGGA-3’ and 5’-GGCGGAAGTAGAATCTCACA-3’ for CCL25, 5’-TGTGTCCGTCGTGGATCTGA-3’ and 5’-TTGCTGTTGAAGTCGCAGGAG-3’ for GAPDH. All primers were purchased from Takara Biotechnology. Samples were run in triplicates, and the mRNA levels of each specific gene were normalized to GAPDH using the comparative Ct method (△△Ct).

### Data analysis

Data are expressed as mean±standard deviation (SD). All statistical analyses were performed using the Prism Graph 6.0 software. Statistical significance of the data was determined using the unpaired, two-tailed Student’s *t*-test. Values of *p*<0.05 were considered statistically significant.

## Results

### 1. Intestinal DCs were altered in their number and maturity in FHF

**Fig 1:**
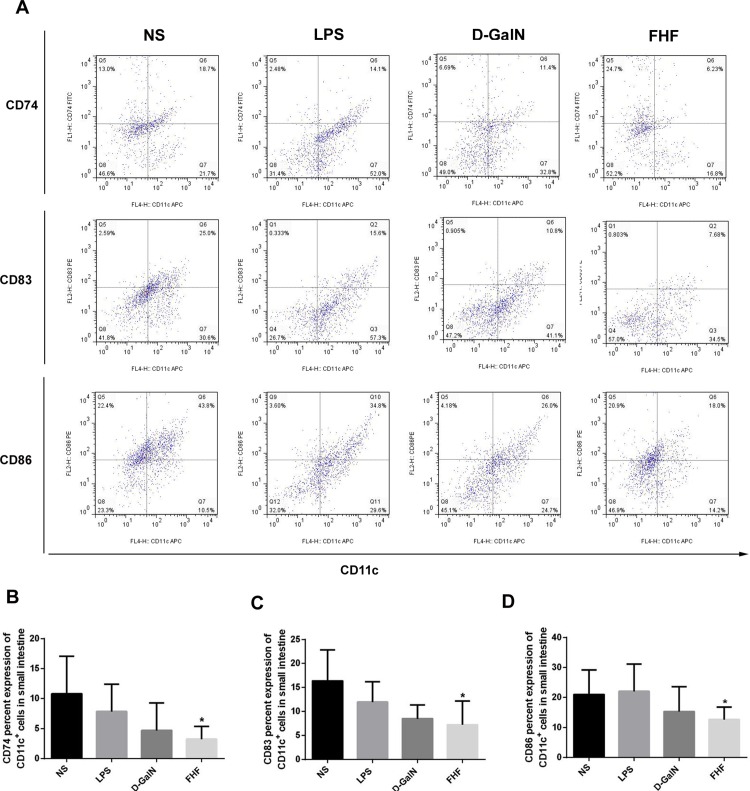
Isolation and phenotypic characterization of intestinal CD11c^+^DCs. (A) Expression of surface markers in MACS-isolated CD11c^+^DCs. (B) Percentage of CD74 in CD11c^+^DCs. (C) Percentage of CD83 in CD11c^+^DCs. (D) Percentage of CD86 in CD11c^+^DCs. Compared with the NS group, expression of CD74, CD83 and CD86 was lower in the D-GalN group and significantly lower in the FHF group, but there was no significant difference compared with the LPS group (*P<0.05 *vs*. the NS group, unpaired *t*-test; *n*≥6).

**Fig 2:**
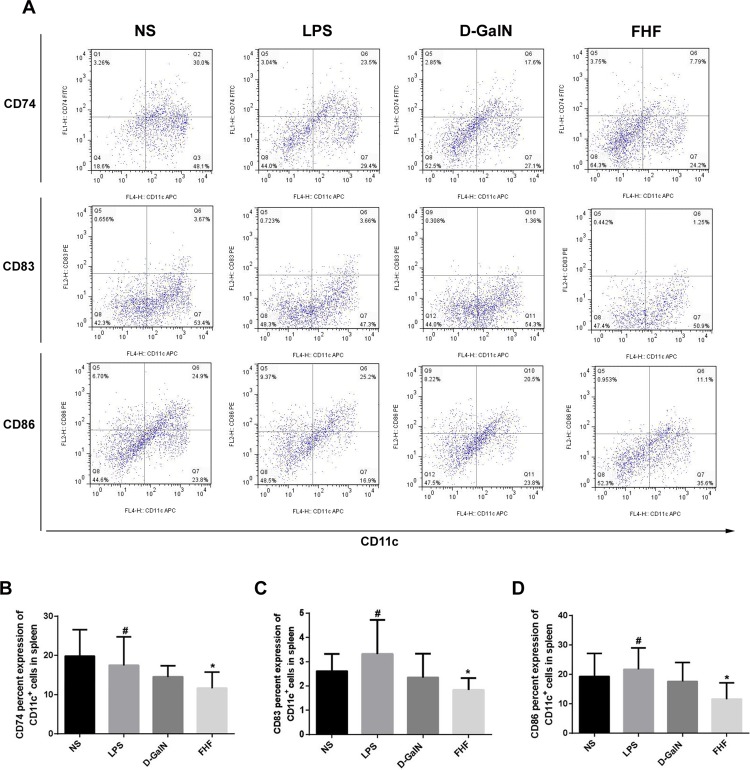
Isolation and phenotypic characterization of spleen CD11c^+^DCs. (A) Expression of surface markers in MACS-isolated CD11c^+^DCs. (B) Percentage of CD74 in spleen CD11c^+^DCs. (C) Percentage of CD83 in spleen CD11c^+^DCs. (D) Percentage of CD86 in spleen CD11c^+^DCs. Compare with the NS group, expression of CD74, CD83 and CD86 in the FHF group was lower in the D-GalN group and significantly lower in the FHF group, but there was no significant difference compared with the LPS group (*P<0.05 *vs*. the NS group, #*P*<0.05 *vs*. the FHF group unpaired t-test; *n*≥6)

CD86 and MHC II are major mature markers of DCs, and CD83 is a transmembrane protein, mainly known as a marker of mature DCs (mDCs) [15]. CD74 is a transmembrane glycoprotein that associates with MHC class II α and β chains and is also known as MHC class II invariant chain (Ii) [16]. The markers CD74, CD83 and CD86 of CD11c^+^DCs were lower in the D-GalN group and significantly lower in the FHF group (*p*<0.05) (**Fig 1**). There was no significant change in CD74, CD83 and CD86 levels in the LPS group (**Fig 1**). Spleen DCs were isolated to reveal any changes of peripheral DCs. Results demonstrated (**Fig 2**) that CD74, CD83 and CD86 of spleen CD11c^+^DCs were lower in the D-GalN group and significantly lower in the FHF group (*p*<0.05), which was consistent with the intestinal DCs.

### 2. The chemotactic ability of intestinal CD11c^+^DCs was altered in FHF

#### 2.1 Immunohistochemical staining of CCR7 and CCR9, along with their ligands CCL21 and CCL25

**Fig 3:**
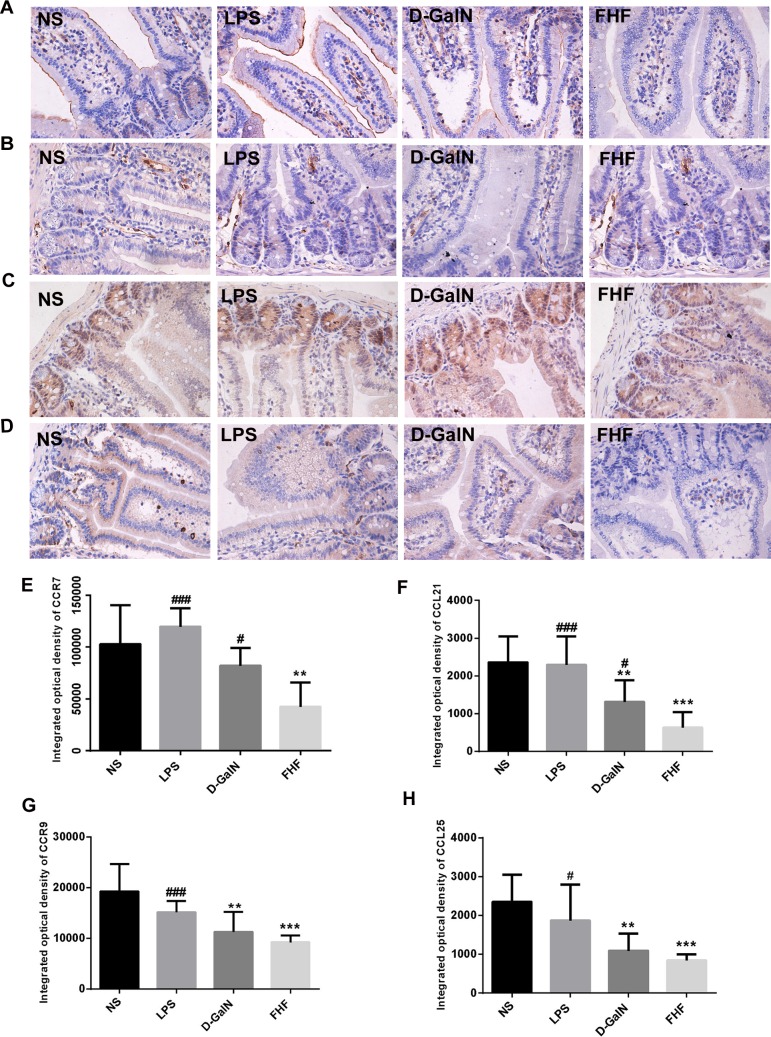
Immunohistochemical staining of CCR7, CCR9, CCL21 and CCL25. A, B, C and D: Immunohistochemical staining of CCR7, CCL21, CCR9 and CCL25; E, F, G and H: Integrated optical density of CCR7, CCL21, CCR9 and CCL25. Compared with the NS group, the integrated optical density of CCR7, CCL21, CCR9 and CCL25 was reduced in the D-GalN group and notably reduced in the FHF group, but there was no significant difference compared with the LPS group (**P< 0.01, ***P< 0.001 *vs*. the NS group; #*P*<0.05, ##*P*<0.01, ###*P*<0.001 *vs*. the FHF group, unpaired *t*-test; *n*≥10).

In the NS group and the LPS group, there was a high intensity of CCR7 (**Fig 3A**) expressed in membranes of intestinal epithelium and lamina propria (LP) cells, indicated by a strong brown. A reduced intensity of CCR7 was observed in the D-GalN group, and in the FHF group there was a significant reduction in CCR7 (*P*<0.01). CCL21 (**Fig 3B**) was strongly expressed in the cytoplasm of LP cells (indicated by brown staining). Expression of CCL21 was lower in the D-GalN group and there was significantly lower in the FHF group compared with the NS group or the LPS group (*P*<0.001). CCR9 (**Fig 3C**) was mainly distributed in the epithelium and LP of the gut. Compared with the NS group and the LPS group, the brown was less intense in the D-GalN group and significantly reduced in the FHF group (*P*<0.001). CCL25 (**Fig 3D**), the ligand of CCR9, was mainly distributed in the intestinal epithelium and its expression was correlated with CCR9, with differences between groups being statistically significant (*P*< 0.001).

#### 2.2 Protein expression of CCR7 and CCR9 as well as their ligands CCL21 and CCL25

**Fig 4:**
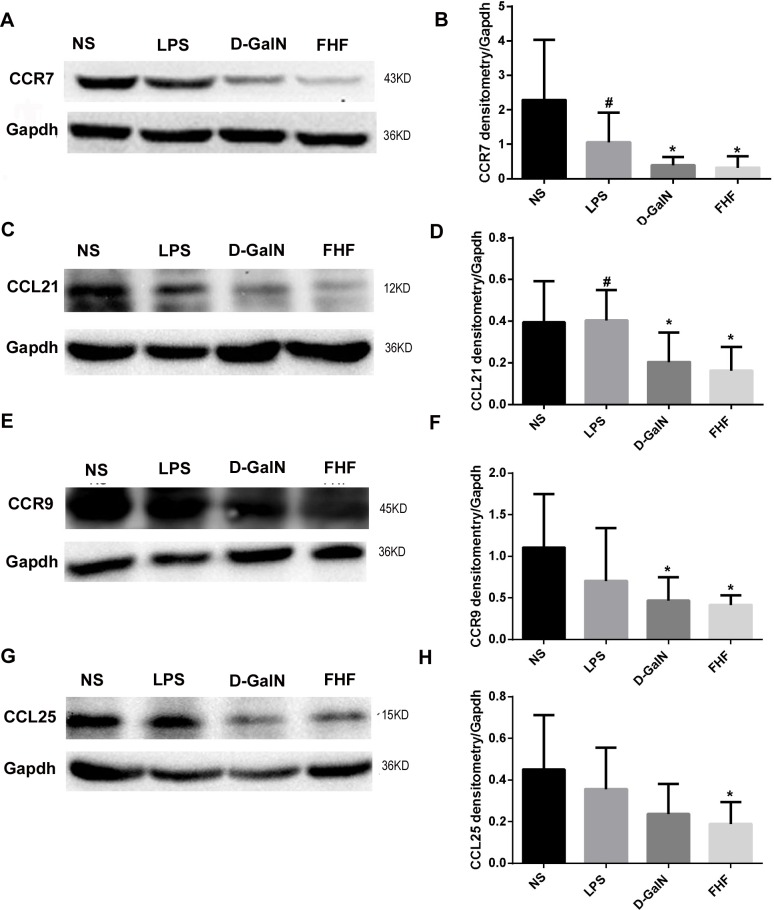
Intestinal CCR7, CCL21, CCR9 and CCL25 protein expression. A, C, E and G: Electrophoresis banding of intestinal CCR7, CCL21, CCR9 and CCL25. B, D, F and H: Densitometric analysis using the Image-Pro software. Compared with the NS group, absorbance ratios of CCR7, CCL21, CCR9 and CCL25 to GAPDH were lower in the D-GalN group and notably decreased in the FHF group, but there was no significant difference compared with the LPS group (*P< 0.05 *vs*. the NS group; #*P*<0.05 *vs*. the FHF group, unpaired *t*-test; *n*≥8).

In the NS group, there were clear and specific bands of CCR7 (**Fig 4A**) and its ligand CCL21 (**Fig 4C**) as well as CCR9 (**Fig 4E**) and its ligand CCL25 (**Fig 4G**) at 43KD, 12KD, 45KD and 15KD, respectively. Expression of these proteins changed little in the LPS group. In the D-GalN group, expression of these proteins was lower compared with the NS group and the LPS group, while in the FHF group protein expression of CCR7, CCL21, CCR9 and CCL25 was significantly decreased (*P*<0.05).

#### 2.3 mRNA expression of CCR7 and CCR9 along with their ligands CCL21 and CCL25

**Fig 5:**
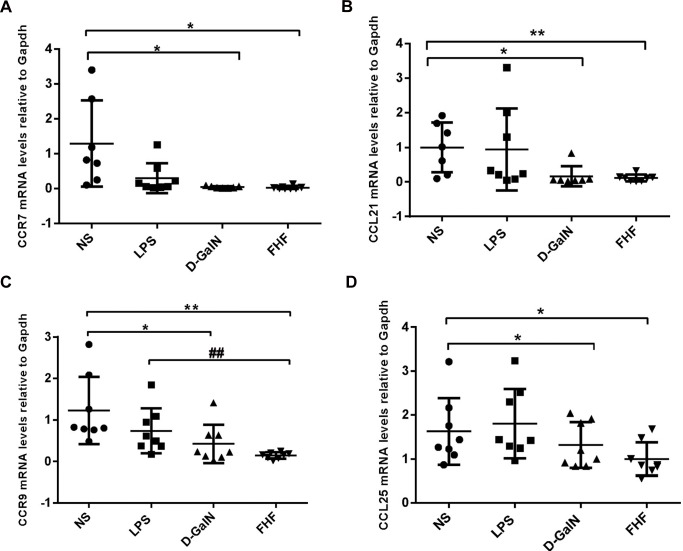
Relative mRNA levels of intestinal CCL21, CCR7, CCR9 and CCL25. (A): CCR7, (B): CCL21, (C): CCR9 and (D): CCL25. Levels of intestinal CCL21, CCR7, CCR9 and CCL25 mRNAs in tissues from the FHF group and D-GalN were significantly decreased compared with the NS group, but there were no significant differences compared with tissues from the LPS group of CCR7, CCR9, CCL21 and CCL25 (*P< 0.05, **P< 0.01 *vs*. the NS group; #*P*<0.05, ##*P*<0.01 *vs*. the FHF group, unpaired *t*-test; *n*≥8).

Compared with the NS group, mRNA expression of CCR7 (**Fig 5A**), CCL21 (**Fig 5B**), CCR9 (**Fig 5C**) and CCL25 (**Fig 5D**) was lower in the D-GalN group and significantly lower (*P*<0.05) in the FHF group. These findings were consistent with the results of immunohistochemical staining and western blot analysis.

## Discussion

Spontaneous bacterial peritonitis (SBP) is a severe complication occurring in patients with FHF. A great deal of intestinal bacterial translocation and over growth, concurrent with increased intestinal epithelial permeability all contribute to SBP [17]. In addition, a dysfunctional intestinal barrier, promoting the translocation of bacteria and bacterial products, plays a key role in SBP.

DCs are efficient antigen-presenting cells (APCs) that stimulate innate and adaptive immune reactions. By interacting with pathogens and presenting antigens to cells of the adaptive immune system, intestinal DCs are key regulators of the intestinal immune barrier and may be involved in FHF complicated with SBP. DCs are distributed widely in the LP and in organized lymphoid organs associated with the intestine, including the ileal Peyer’s patches (PPs), colonic isolated lymphoid follicles (ILFs) and the more remote MLNs [18]. Different DC subsets have been described in mice and human classified on the expression of their surface makers. Generally, mouse DCs express CD11c, CD11b, the interdigitating DC marker CD205, and the co-stimulator molecules CD80, CD86 and CD40 [19]. CD74, which is MHC class II-associated invariant chain (Ii), parallels the tissue distribution of MHC class II [20]. CD74 has been reported to regulate DC migration [21]. In our study, CD11c, CD74, CD83 and CD86 in both intestinal DCs and spleen DCs were significantly reduced. This showed that the number of DCs and their maturation process were disrupted in FHF.

Intestinal DCs can be divided into resident DCs and migratory DCs. Presenting antigens to naïve T cells and initiation of adaptive immune responses are tightly associated with DC migrating property. DC migration occurs constitutively but is markedly accelerated after infection or inflammation [22]. Gene targeting has shown that CCR7 is essential for DC mobilization [23], and has been established as the critical receptor for entry of DCs into lymph nodes, but is now also known to be important for the mobilization of DCs [24]. CCR7 is a G-protein-coupled receptor and mediate signaling by seven trans-membrane segments [25, 26]. CCR7 is expressed by various subtypes of immune cells including semi-mature and mature DCs[27], T cells [28] and B cells [29]. CCL19 and CCL21, also known as EBI1-Ligand Chemokine/Macrophage Inflammatory Protein-3b (ELC/MIP-3b) and Secondary Lymphoid-tissue Chemokine (SLC), are the only two well-characterized CCR7 ligands mainly expressed in secondary lymphoid tissues [30]. Recent evidence suggested that CCL19 and CCL21 were able to drive the migration of DCs, as well as directly affecting their ability to prime T cells [31]. It had also been shown that CCR7 ligands increased the antigen uptake of mature DCs [32]. Though binding to CCR7 with similar affinity, CCL19 and CCL21 have distinct regulatory effects of CCR7. CCL19 promotes the receptor phosphorylation and desensitization [33], while CCL21 has a unique C-terminal extension that binds glycosaminoglycans (GAGs) to trigger directed migration [24]. Mirjam R *et al*. reported that CCL21 was sufficient to mediate DC migration, maturation and T cell priming in the absence of CCL19 [34].

In order to further investigate the change of chemotactic ability in DCs with FHF, we detected a CCR9/CCL25 axis in intestinal tissues. CCR9/CCL25 interaction is a critical chemokine/receptor pairs involved in gut-specific migration of leukocytes and lymphocyte recruitment to the intestine. CCL25 is expressed in thymus and small intestinal epithelium [35] and regulates trafficking of gut tropic effect or T cells via upregulation of the integrin homing receptor α4β7, which interacts with MadCAM-1 on intestinal microvascular endothelium [36, 37]. Accumulating evidence had shown that the CCR9/CCL25 axis participated in a variety of disease processes [38, 39]. The levels of colonic CCL25 had been shown to increase in both Crohn’s disease and ulcerative colitis, and pharmacological inhibitors of CCR9 prevented the development of ulcerative colitis in an experimental model [40]. Our results confirmed that, consistenting with CCR7 and its ligand CCL21, expression of CCR9 and its ligand CCL25 were reduced in intestinal tissues in the D-GalN group and significantly reduced in the FHF group. Decreased expression of CCR7/CCL21 and CCR9/CCL25 demonstrated a reduced chemotactic ability of intestinal DCs in FHF.

In conclusion, we had shown that CD11c^+^DCs not only decreased in number and maturity, but also in chemotactic and migration ability in FHF. The maturation process of DCs in the intestinal tract was obstructed, and the migration and homing property of DCs was restricted, which affected the function of the intestinal immune barrier in FHF. These findings increase our understanding of the mechanisms involved in FHF concurrent with SBP, and may open up therapeutic opportunities for FHF complicated with SBP.

## Supporting Information

S1 File Raw data of the experiments. The minimal raw data of Flow cytometry, Immunohistochemistry, Western Blot and Real-time PCR analysis.
(RAR)
